# A case report of adult rectal duplication cyst resected by synchronous trans-abdominal and trans-anal total mesorectal excision

**DOI:** 10.1016/j.ijscr.2020.07.058

**Published:** 2020-07-24

**Authors:** Tetsuro Tominaga, Takashi Nonaka, Akiko Fukuda, Masaaki Moriyama, Syouzaburou Oyama, Shigekazu Hidaka, Kazuhiro Tabata, Terumitsu Sawai, Takeshi Nagayasu

**Affiliations:** a Department of Surgical Oncology, Nagasaki University Graduate School of Biomedical Science, 1-7-1 Sakamoto, Nagasaki 852-8501, Japan; b Department of Pathology, Nagasaki University Graduate School of Biomedical Science, 1-7-1 Sakamoto, Nagasaki 852-8501, Japan; c Department of Cardiopulmonary Rehabilitation Science, Nagasaki University Graduate School of Biomedical Science, 1-7-1 Sakamoto, Nagasaki 852-8501, Japan

**Keywords:** ta-TME, trans-anal total mesorectal excision, Duplication cyst, Rectum, Trans anal approach, Case report

## Abstract

**Introduction:**

Rectal duplication cyst is extremely rare. Most of the cases diagnosed before two years old. Adult onset rectal duplication cyst usually contains malignant formation. Then complete tumor resection is needed for the disease to prevent malignant change.

**Presentation of case:**

A 52-year-old man was referred to our hospital due to bloody stool. Colonoscopy showed 20-mm of sub-mucosal tumor at low rectum. Biopsy revealed no specific findings. Abdominal computed tomography showed that a 20-mm low density mass at posterior of lower rectum. As the existence of malignant lesion was unassailable, we planned two team, trans abdominal and trans-anal, laparoscopic low anterior resection. Perioperative course was good. Macroscopically, rectum had a submucosal cystic mass fulfilled mucinous content. Histologically, cyst wall was covered with heterotopic ciliated epithelium and composed of smooth muscle.

**Discussion:**

Trans-anal total mesorectal excision (ta-TME) was introduced as beneficial approach for rectal malignancies.

**Conclusion:**

Complete resection was possible by TME was necessary for patients with adult rectal duplication cyst, and synchronous trans-abdominal and trans-anal approach is a promising option.

## Highlights

• Rectal duplication cyst is extremely rare.
• Adult onset rectal duplication cyst usually contains malignant formation.
• Complete tumor resection is needed for the disease to prevent malignant change.
• Synchronous trans abdominal and anal approach was effective for complete tumor resection.

## 1 Introduction

Rectal duplication cyst is rare congenital malformation, and the disease is commonly diagnosed before two years old [1,2]. Some previous reports showed that “adult” rectal duplication cyst usually contains malignant formation [3,4]. Then management of adult rectal duplication cyst includes surgical resection to prevent malignant change [3].

Trans-anal total mesorectal excision (ta-TME) was introduced as beneficial approach for low rectal cancer [5,6]. The approach preformed from lower edge of tumor by direct visualization, keep a safe distal margin, as mesorectal excision could be completed [7]. However, there were limited reports for TME or trans anal TME for rectal duplication cyst [3,8].

In this case report, we described adult rectal duplication cyst resected by ta-TME.

This work has been reported in line with the SCARE criteria [9].

## 2 Case presentation

**Fig. 1:**
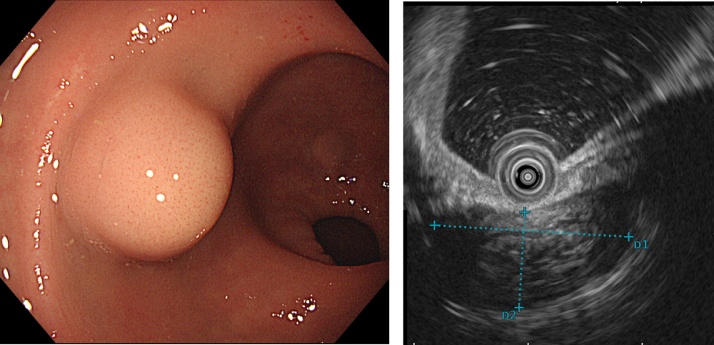
Colonoscopy finding. Colonoscopy showed 20-mm of sub-mucosal tumor at low rectum (Fig. 1a). The tumor was elastic hard and move easily. An endoscopic ultrasonography revealed low echoic mass (Fig. 1b). Biopsy revealed no specific findings.

**Fig. 2:**
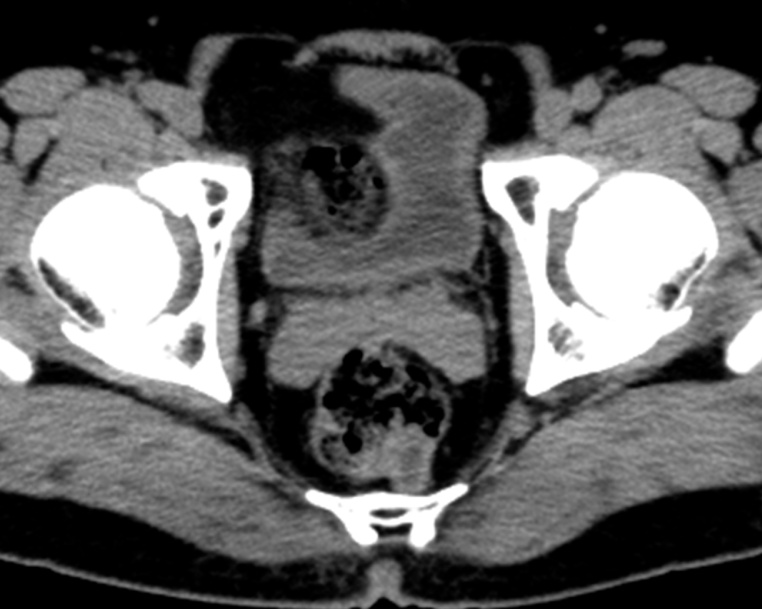
Pelvic CT finding. Pelvic CT showed that a 20-mm low density mass at posterior of lower rectum.

A 52-year-old man was referred to our hospital due to bloody stool. He received medication due to schizophrenia. He had no allergies, family history, and surgical history. His vital sign was stable. Physical examination demonstrated no abdominal pain. On digital examination, there was a smooth rubbery mass located 6 cm from the anal verge. No inflammation and anemia were observed by laboratory data. His tumor markers including carcinoembryonic antigen and cancer antigen19-9 were not elevated. Colonoscopy showed 20-mm of sub-mucosal tumor at low rectum (Fig. 1a). The tumor was soft and move easily. An endoscopic ultrasonography revealed low echoic mass (Fig. 1b). Biopsy and repeat boring biopsy revealed no specific findings. Abdominal computed tomography showed that a 20-mm low density mass at posterior of lower rectum (Fig. 2). There was no swollen lymph node surround the tumor. Differential diagnoses were dermoid cyst, enteric cyst, hamartoma, teratoma, neuroendocrine tumor, gastrointestinal stromal tumor, and duplication cyst. As the existence of malignant lesion was unassailable, we obtained informed consent from the patient. Then we planned TME for synchronous trans abdominal and trans-anal approach.

**Fig. 3:**
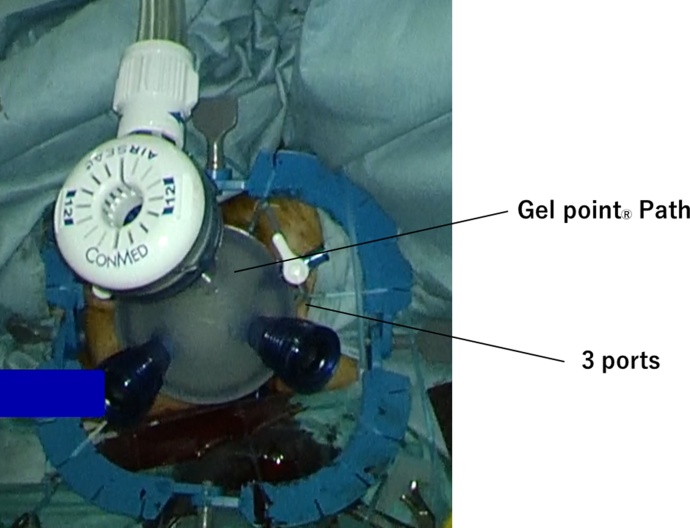
Intra-operative finding for ta-TME. we introduced GelPOINT^R^ Path Trans anal Access Platform (Applied Medical, Inc. Rancho Santa Margerita, CA, USA) to anal canal. A standard 10 mm-camera and conventional laparoscopic instrument were used.

**Fig. 4:**
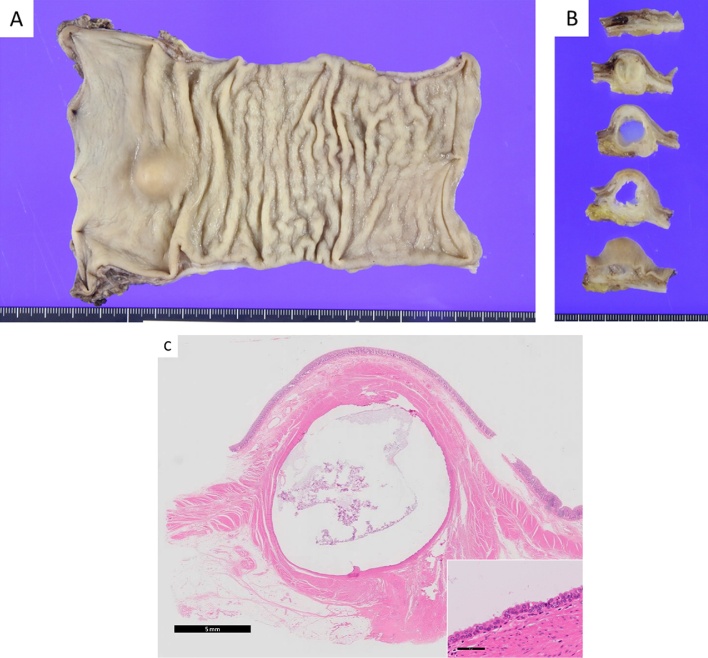
Macroscopic and histological appearance. a) Macroscopic finding of resected rectum, b) the cyst was fulfilled by mucinous content. c) Histologically, the cyst was composed of single epithelial layer and smooth muscle layer. In high magnification, epithelial layer was ectopic ciliated epithelium.

Two surgeon team, abdominal and trans-anal team, performed the operation to shorten operation time, reduce blood loss and recognize proper layer. Each surgeons had ten-year experience in gastrointestinal surgery. Abdominal surgery performed laparoscopically using 5 port technique (10 mm port above the umbilicus, 5 mm port in the right and left upper/lower quadrant). They transected inferior mesenteric artery and vein. Then splenic flexure was full mobilized. To perform ta-TME, we introduced GelPOINT^R^ Path Trans anal Access Platform (Applied Medical, Inc. Rancho Santa Margerita, CA, USA) to anal canal. A standard 10 mm-camera and conventional laparoscopic instrument were used (Fig. 3). The tumor located low to middle rectum. The rectal lumen was double purse-string closed with prolene suture. Starting posteriority, dissection was carried out through rectal wall to the mesorectal fascia, and up to presacral space. Next, full thick ness incision anterior wall to lateral wall. When we perform lateral dissection, we mind not to injury sacral nerve and neurovascular bundle to prevent postoperative urological disorder. Finally, the anal space communicated to abdominal space. The specimen was removal through the enlarged umbilicus wound. The reconstruction was completed by single stapling technique. Macroscopically, rectum had a submucosal mass sized 15 mm in diameter (Fig. 4a) that showed cystic change fulfilled mucinous content in cutting surface (Fig. 4b). Histologically, cyst wall was covered with heterotopic ciliated epithelium and composed of smooth muscle. Finally, rectal duplication cyst was diagnosed (Fig. 4c). Fortunately, the tumor was completely resected, and there was no evidence of malignant change. Postoperative course was uneventful. After the six months clinical follow up with no adjuvant treatment, he has no evidence of recurrence.

## 3 Discussion

Rectal cystic lesions are rare condition which include epidermoid cyst, dermoid cyst, enteric cyst, rectal cystic hamartoma, teratoma and duplication cyst[10]. The features of duplication cyst are usually single, tubular or cystic tumor, and located at mesenteric side. Most of the patients with duplication cysts were under two years old, and one-third of the patients was neonate [11]. Previous report examining 398 patients with duplication cyst showed that 53% of lesion was located at small intestine, and only 4.5% of them was located at rectum [4]. The clinical symptoms was depending on tumor location, and it has been estimated that almost 50% of cases are asymptomatic [12]. Rectal duplication cysts often developed bloody stool, constipation, feeling rectal fullness, and lower abdominal pain. The main complain of this adult patient was continuous bloody stool.

Histopathological feature of this disease contains of a following congenital anomalies: the presence of smooth muscle and epithelial lining, and both structures closely attached to gastrointestinal tract and sharing a common wall [13,14]. In the present case, the cyst was covered with ectopic ciliated epithelium and smooth muscle layer.

Most of rectal duplication cysts were benign. However, some previous reports showed malignant changes especially for adult patients [3,14]. Recently, complete TME with adequate lymph node dissection is a standard approach for rectal malignancies to prevent tumor spread and local recurrence [[15], [16], [17]]. Then complete TME is needed for adult onset rectal duplication cyst to prevent the potential risk of recurrence and malignant change.

However, TME sometimes could not be success due to narrow pelvis, obese, and large tumor [5]. Moreover, surgical resection of sometimes difficult because of anatomical site and dealings: posteriority the sacral vessels; laterally the hypogastric plexus and its branch; anteriority the prostate or vagina [18]. Several approaches were reported by trans abdominal, trans-anal, trans coccygeal, and posterior sagittal approach [12,19]. In this case, we performed TME for synchronous trans abdominal and trans-anal approach by two surgeon team. The lower edge of tumor was visible directly by trans anal approach and could keep adequate distal margin [7]. Additionally, multidirectional view from two surgeon team enable to recognize proper dissection layer, which leads complete TME and avoid injury of surrounding tissue [20].

## 4 Conclusion

Complete resection was possible by TME was necessary for patients with adult rectal duplication cyst, and synchronous trans-abdominal and trans-anal approach is a promising options.

## Declaration of Competing Interest

None.

## Funding

None.

## Ethical approval

None.

## Consent

Written informed consent was obtained from the patient for publication of this case report and accompanying images. A copy of the written consent is available for review by the Editor-in-Chief of this journal on request.

## Author contribution

Tetsuro Tominaga and Takashi Nonaka conceptualized the study. Akiko Fukuda, Masaaki Moriyama, Syouzaburou Oyama, and Shigekazu Hidaka collaborated in the patient’s care. Kazuhiro Tabata made a pathological diagnosis.Terumitsu Sawai and Takeshi Nagayasu provided input on the manuscript.

## Registration of research studies

This case report does not correspond to “first in man study” i.e. the first time a new device or surgical technique.

## Guarantor

Tetsuro Tominaga

Department of Surgical Oncology

Nagasaki University Graduate School of Biological Sciences

1-7-1 Sakamoto, Nagasaki 852–8501, Japan

Phone: +81−95-819−7304 Fax: +81−95-819−7306

E-mail: tetsuro.tominaga@nagasaki-u.ac.jp

## Provenance and peer review

Not commissioned, externally peer-reviewed.
